# Dynamics of the Methanogenic Archaea in Tropical Estuarine Sediments

**DOI:** 10.1155/2013/582646

**Published:** 2013-01-17

**Authors:** María del Rocío Torres-Alvarado, Francisco José Fernández, Florina Ramírez Vives, Francisco Varona-Cordero

**Affiliations:** ^1^Department of Hydrobiology, Universidad Autónoma Metropolitana-Iztapalapa, Avenida San Rafael Atlixco No. 86, Colonia Vicentina, 09340 Mexico City, DF, Mexico; ^2^Department of Biotechnology, Universidad Autónoma Metropolitana-Iztapalapa, Avenida San Rafael Atlixco No. 86, Colonia Vicentina, 09340 Mexico City, DF, Mexico

## Abstract

Methanogenesis may represent a key process in the terminal phases of anaerobic organic matter mineralization in sediments of coastal lagoons. The aim of the present work was to study the temporal and spatial dynamics of methanogenic archaea in sediments of tropical coastal lagoons and their relationship with environmental changes in order to determine how these influence methanogenic community. Sediment samples were collected during the dry (February, May, and early June) and rainy seasons (July, October, and November). Microbiological analysis included the quantification of viable methanogenic archaea (MA) with three substrates and the evaluation of kinetic activity from acetate in the presence and absence of sulfate. The environmental variables assessed were temperature, pH, Eh, salinity, sulfate, solids content, organic carbon, and carbohydrates. MA abundance was significantly higher in the rainy season (10^6^–10^7^ cells/g) compared with the dry season (10^4^–10^6^ cells/g), with methanol as an important substrate. At spatial level, MA were detected in the two layers analyzed, and no important variations were observed either in MA abundance or activity. Salinity, sulfate, solids, organic carbon, and Eh were the environmental variables related to methanogenic community. A conceptual model is proposed to explain the dynamics of the MA.

## 1. Introduction

Coastal and marine environments, including estuaries and coastal lagoons, are characterized by large amounts of organic matter, which is mineralized primarily in sediments through anaerobic processes, sulfate reduction being the dominant metabolic pathway [1, 2]. However, although these ecosystems are the typical habitat of sulfate-reducing prokaryotes (SRP), methanogenic archaea (MA) and methane production have also been detected [3, 4].

MA are strict anaerobes that produce methane as end-product of their metabolism. These organisms are common in anoxic environments in which electron acceptors such as nitrate and sulfate are either absents or present at low concentrations and are usually dominant in freshwater environments. In the presence of these electron acceptors, methanogenesis is outcompeted by anaerobic respiration, mainly for thermodynamic reasons [5]. MA distribution patterns and its number, as well as physical, chemical, and nutritional parameters controlling their abundance and distribution have been studied in lacustrine sediments [6] and in coastal environments [7, 8]. 

Most of the ecological studies assessing the structure of methanogenic communities in estuarine systems have been performed in temperate latitudes where temperature is one of the major factors regulating ecosystem function. These investigations have included an evaluation of the MA in the intertidal zone of marshes with the presence of *Spartina alterniflora*, whose roots provide organic carbon and contribute to create aerobic microhabitats [9, 10]. MA abundance has been quantified with two or three substrates, of which acetate and hydrogen have been reported as the two most important ones [4, 11]. Additionally, it has been established that in estuaries, where a salinity gradient exists from the marine zone to a river entrance, MA are prevalent upstream in the freshwater region and decrease towards the brackish and marine ends; sulfate reduction has been identified as the key factor related to the MA distribution [7, 10, 12, 13]. Depth profiles of MA distribution have been observed, their abundance increase in deeper layers of the sediment column, because the MA are dependent on heterotrophs and fermenters during the organic matter decomposition, its decline is also related to a decrease in both sulfate concentration and redox potential [8]. 

In contrast to estuaries, coastal lagoons generally have restricted communication with the sea and in tropical lagoons, as a result of strong seasonal precipitation patterns, there are significant fluctuations in river discharge, and associated hydrological conditions (salinity). These variations might affect the structure of microbial communities involved in the terminal phases of the anaerobic organic matter mineralization, as well as to the biogeochemical processes related to it. In spite of its importance, studies focused on these ecosystems to assess the dynamics of anaerobic microbiota, especially MA, are scarce. It has been reported that MA using methylamines are the primary microbial components in sediments of coastal lagoons associated to mangroves, with higher densities during the summer and premonsoon [14, 15]. In another study, a peak of methane production in mangrove sediments has been recorded in the postmonsoon season [16]. In Mexico, where coastal lagoons are abundant, investigations on methanogenic communities are virtually absent; hence, the aim of the present study was to explore the spatial and temporal dynamics of the methanogenic community in sediments from two tropical coastal systems: Chantuto-Panzacola and Carretas-Pereyra, located in the Mexican southern Pacific and to propose a conceptual model on MA dynamics in sediments for the tropical coastal lagoons studied.

## 2. Materials and Methods

### 2.1. Study Site

**Figure 1:**
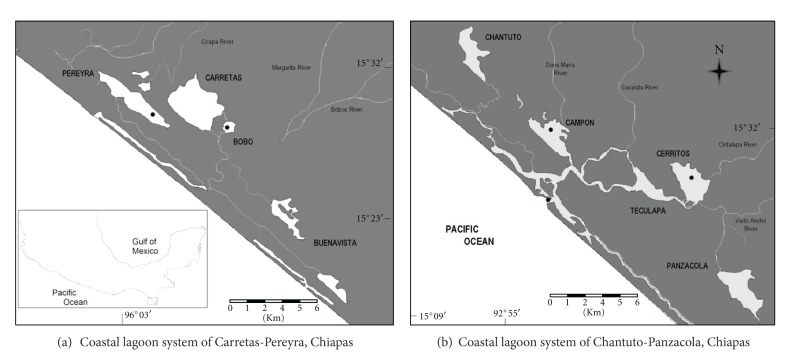
Study area and sampling sites (●).

The Chantuto-Panzacola and Carretas-Pereyra lagoon systems are located in the State of Chiapas, Mexican Pacific coast (Figure 1); they are part of the International Biosphere Reserve “La Encrucijada”. The climate of the region is warm (28°C) and humid (89%) with abundant summer rainfall; annual rainfall ranges between 1,300 and 3,000 mm. The rainy season begins between May and June and continues through November; the dry season occurs from December to May [17]. Lagoon systems are characterized by high temperatures in the water column (29–35.5°C), with a variable salinity ranging from 0 to 34.5‰  in Chantuto-Panzacola and from 0 to 22.7‰  in Carretas-Pereyra, depending on the season. There is a limited exchange with the sea and a significant phosphorus supply from rivers, which favors high chlorophyll-*a *levels. Systems are bordered by mangrove forests and freshwater wetlands. Mangrove detritus results in high humic substance levels (>150 mg/L) in the rainy season [18] also recording high ammonium concentrations derived from mineralization [19]. 

The Chantuto-Panzacola lagoon has an area of 18,000 ha and comprises five lagoons: Chantuto, Campón, Teculapa, Cerritos, and Panzacola. In this system, samples were collected from the Cerritos and Campón lagoons (Figure 1). The Cerritos lagoon (15°09′54.4′′N, 92°45′34.0′′W) has a mean depth of 1.1 m in the dry season and 1.3 m during the rainy season. The Cintalapa River flows into this lagoon, contributing a volume between 66.2 m^3^/s in October and 0.4 m^3^/s in May (dates proportionated by the National Water Commission in Mexico). The Campon lagoon (15°12′30.0′′N, 92°51′24.2′′W) has a mean depth of 0.8 m in the dry season and of 0.9 m in the rainy season. The Cacaluta River flows into this lagoon, with a maximum inflow in October (144.2 m^3^/s) and a minimum inflow in May (0.5 m^3^/s). Sediments are a mixture of silt and sand in both lagoons. The Carretas-Pereyra system covers an area of 3,696 ha and comprises four water bodies: Pereyra, Carretas, Bobo, and Buenavista, sampling took place in Pereyra and Bobo (Figure 1). The Pereyra lagoon (15°31′26.1′′N, 92°51′24.2′′W) has a mean depth of 0.7 m in the dry season and 1.0 m in the rainy season. Sediment is silt-sand. The Margaritas River drains into the Pereyra lagoon (discharge volume unknown). The Bobo lagoon (15°29′22.0′′N, 93°08′44.6′′W) has a mean depth of 0.5 m and 0.7 m in the dry and rainy seasons, respectively. It lacks freshwater inputs and sediment is silt-sand.

### 2.2. Sample Collection and Preparation Procedures

Sediment cores were collected with a 45 cm long and 4.5 cm wide plexiglass coring device during the dry (February, May, and early June) and rainy seasons (July, October, and November). Temperature, Eh, and pH were simultaneously measured when sampling the cores at two sediment depths (6 and 12 cm) using standard electrodes and an Ionanalizer (Conductronic pH 120). pH was measured with a glass electrode and the sediment redox potential was measured using a platinum electrode and a saturated KCl calomel reference electrode (Instrulab, Mexico). The standard potential of the reference (+198) was added to the mean value to obtain the Eh of the sediment medium. Electrodes were routinely standardized in the field using a ZoBell Solution [20]. Subsequently, samples were transported to the laboratory.

Cores obtained in each sampling station were segmented in two sections (0–6 cm and 6–12 cm) under a nitrogen atmosphere. After each section was homogenized in a plastic bag using steady shaking, subsamples were immediately taken to quantify MA. The remaining sediment was maintained under low temperature to perform physical-chemical analyses. 

### 2.3. Microbiological Analyses

Enumeration of viable MA was performed using the Most Probable Number (MPN) method by a ten-fold dilution series (10^−1^ to 10^−10^) for each sample using four tubes per dilution. The MPN analyses included the quantification with substrates commonly used by the different groups of MA: acetate, CO_2_ + H_2_, and methanol, with the basic medium by Balch et al. [21]. Salinity in the culture medium was adjusted with a NaCl (330 g/L) solution to obtain similar values to those measured in the original sediment sample; the pH was adjusted to 7.2 with a bicarbonate (10%) solution. Cultures were incubated at 32°C for one month. Methane was detected with a GOW-MAC Series 580 GC with a thermal conductivity detector (TCD) under the following operation conditions: column, detector, and injector temperatures of 140, 190, and 170°C, respectively; 25°C/min rate; column packed with carbosphere 80/100, helium as carrier gas at 25 mL/min; polarity of 120 mA.

In order to determine the effect of sulfate on MA for a competitive substrate, methanogenic activity was determined in a medium without sulfates (sulfate-free), using 125-mL serum bottles, with 42 mL of the Balch et al. [21] and acetate as substrate to a final concentration of 20 mM. Experiments were conducted in parallel in which the culture medium was supplemented with sulfate (final concentration 20 mM). Bottles were inoculated with 8 mL of moist sediment and incubated at 32°C in the dark for 42 days; the incubations were shaken three times per week. Each experiment was run by duplicate for each sample, including the respective controls (without acetate), with and without sulfates in the medium. Mineralization was evaluated by determining changes in acetate concentration and percent methane production in bottles. For acetate analysis, 1.5 mL samples were centrifuged at 1,120 gf for 10 min. The supernatant was filtered. A 950 *μ*L aliquot was acidified with 50 *μ*L of HCl (2.2 M). The acetate concentration was measured by flame ionization gas chromatography (Agilent Series 6890 Plus) using an Agilent crosslinked FFAP capillary column (15 m × 0.530 mm × 1.00 *μ*m). Column, injection port, and FID temperatures were 120, 130, and 150°C, respectively. The temperature of the column, detector, and injector were 120, 150, and 130°C, respectively. The carrier gas was N_2_ (4.5 mL/min).

### 2.4. Physicochemical Analyses

Sediment samples were centrifuged at 1,602.76 gf at low temperature (4-5°C) for 20 minutes to separate porewater from sediments [22]. Porewater was filtered through 0.45 *μ*m Millipore membranes and the following parameters were determined: salinity, with an optical refractometer (American Optical); sulfate [23] and total dissolved carbohydrates, with the phenol-sulfuric acid technique [24]. Total solids and volatile solids were quantified in moist sediments [25], porosity was determinate by measuring the weight loss by drying sediment samples of know volumes and weights. Organic carbon content was measured through the method by Gaudette et al. [26] in a sediment sample dried at 60°C.

### 2.5. Statistical Analyses

The data matrix included MA abundances and physicochemical variables. To meet the normality assumptions, data for variables were transformed through log⁡*x* + 1 [27]. For the temporal analysis, variables were grouped into two climate seasons (dry and rainy); for the spatial analysis, data were grouped into two depth categories (0–6 cm and 6–12 cm). An analysis of variance (ANOVA) was conducted to test for significant differences between seasons in each system, on the one hand, and between depth categories, on the other. The significance of specific differences was assessed through the Tukey-Kramer multiple comparison test [27]. A Canonical Correspondence Analysis (CCA) was used to investigate the relationship between microbial abundance and environmental variables [28]. These analyses were conducted with the Statistica 10 (Academic) and MVSP 3.12b Software.

## 3. Results and Discussion

The aim of this study was to analyze the changes in the abundance and activity of MA and relate these community characteristics with some physicochemical variables to propose a conceptual model of methanogenic community dynamics in coastal lagoon sediments.

### 3.1. Environmental Variables

Conditions in the sedimentary habitat in the Chantuto-Panzacola and Carretas-Pereyra lagoon systems resulted from seasonal variations between the dry and rainy seasons. Temperature in the sediment was higher in the dry season in comparison with rainy season (Table 1); the temporal variations were significant in Chantuto-Panzacola (Table 2). Significant differences in pH were observed (Table 2). In the dry season, a greater marine influence favors neutral conditions; by contrast, in the rainy season the higher fluvial inflow decreased marine influence, and acid conditions were registered (Table 1). The redox conditions were similar to those reported for sediments from mangroves [29] and were significantly less reductive in the rainy season (Table 1) when the freshwater inflow favored sediment suspension in the water column (turbidity = 126–224 NTU), with an increase in porosity and less reduced conditions at the sediments. In the dry season redox potential decreased as a result of sediment deposition (turbidity = 31–107 NTU). 

The major changes were determined in salinity and sulfate content (Tables 1 and 2). Maximum values were recorded in the dry season and minimum in the rainy season; even totally freshwater conditions existed in both systems in October (0‰). The decrease in salinity and sulfates was due to an increase in fluvial inflow and precipitation. Salinity in coastal lagoons varies according to annual cycles, which depend on the local climate, continental freshwater runoff, connection with the sea, and influence of tides. Knoppers and Kjerfve [30] point out that seasonal pulses in freshwater inflow exert a marked impact on the ecology of coastal lagoons, besides controlling salinity, increasing the water level, and holding open communication to the sea. 

No significant temporal variations were observed in the concentration of total solids and organic fractions (volatile solids, organic matter, organic carbon, and carbohydrates) (*P* > 0.05); and their supply was constant through rivers and wetlands. The high rate of freshwater inflow with organic debris from land and run-off as well as from adjacent mangroves is a key factor related to the contribution of organic matter in coastal zones [31]. 

Spatially there was no pattern of physicochemical conditions in the sedimentary habitat as evidenced by the null significance observed for the temperature, pH, salinity and sulfates (*P* > 0.05). An exception was the Eh, which decreased significantly with depth (Tables 1 and 2). The vertical fluctuations in Eh may be attributed to a reduction in the oxygen diffusion rate in porewater as the depth of the sediment column increases [32]. There were no significant variations in solids content and organic fractions (*P* > 0.05) (Table 1). However the organic carbon content was higher in the sediment layer of 12 cm, dos Santos Fonseca et al. [33] point out that this behavior seems to result from the fact that the most labile substrate is readily used by the microbial community in the top centimeters of sediment, and the refractory fraction builds up in deeper layers, where it will be degraded slowly. The presence of refractory material (wood and phytoplankton debris identified with a light microscope Zeiss Axioscop) concentrated largely in the 6–12 cm-deep layer in Pereyra and Campón lagoons seem to support this hypothesis.

### 3.2. Abundance and Distribution of MA

**Figure 2:**
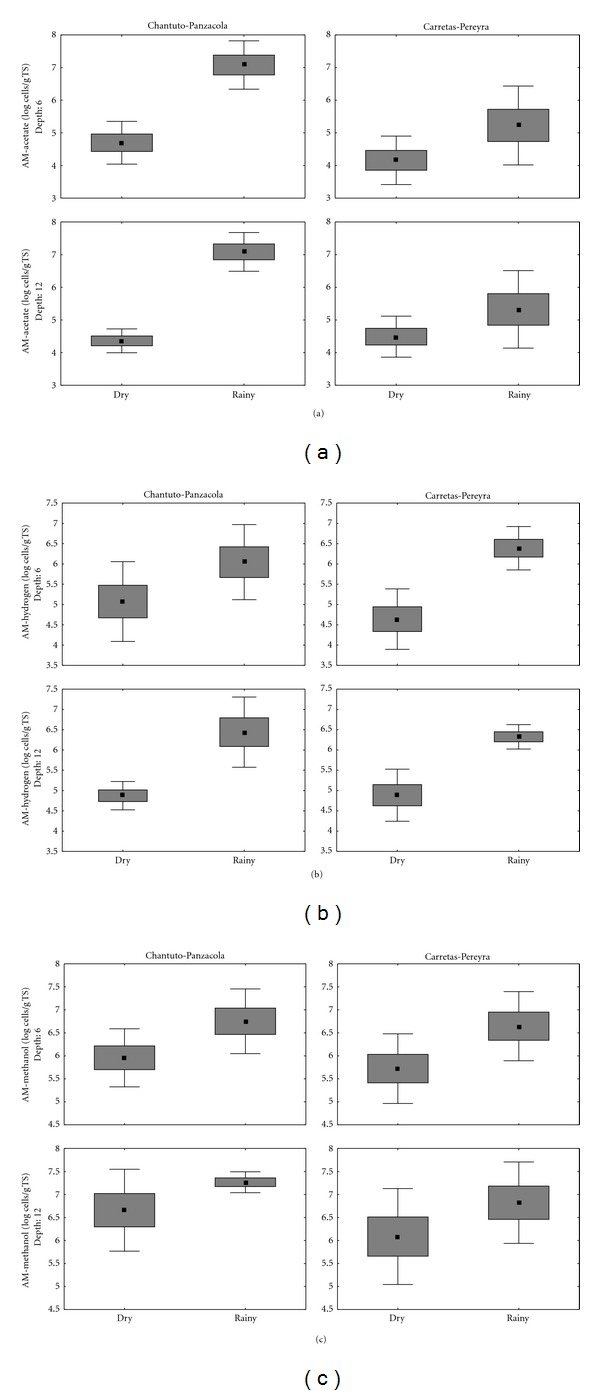
Temporal and spatial variation in the abundance of MA (log cells/g TS).

Viable MA in the sediments of Chantuto-Panzacola and Carretas-Pereyra systems were evaluated with MPN, obtaining a range of abundance between 10^4^ and 10^7^ cells/g. MA density reached peak levels in the rainy season, with a significant decrease of as much as two orders of magnitude during the dry season (*P* < 0.05) (Figures 2(a)–2(c)). In the rainy season, increased freshwater input created favorable conditions for MA proliferation. In this season highest levels of MA were recorded with acetate and methanol in Chantuto-Panzacola and with methanol and H_2_-CO_2_ in Carretas-Pereyra. During the dry season, high MA levels were obtained with methanol in both lagoon systems; the second substrate in importance was H_2_-CO_2_ and the lowest levels correspond to acetate (Table 3). 

The constant occurrence of MA was probably the result of their ability to use different electron donors in an ecosystem with a constant supply of organic matter provided by the rivers and run-off from adjacent mangroves. Verma et al. [34] mentioned that the continued presence of MA in coastal lagoons is possible by the presence of “noncompetitive” substrates, (methanol and methylamines), that are used exclusively by the MA, as well as the constant availability of “competitive” substrates (acetate and hydrogen), used by methanogen and other anaerobic microorganisms.

Methanol was an important substrate in both seasons, may be released from methoxy groups during degradation of lignin. Methanol-utilizing MA have a broad substrate spectrum, can also grow on acetate, growth on H_2_-CO_2_ is restricted to some *Methanosarcina* species [5]. There is evidence supporting the hypothesis that cometabolism of a broad range of substrates by generalist microorganisms may confer competitive advantages [35]. Purdy et al. [13] mention that, within the methanogenic community, the presence of generalist groups implies that these are better adapted to the variations in the estuarine conditions. Additionally methanol allows MA to maintain their populations in the presence of sulfate, which act favoring sulfate reduction. The key role of other methylated compounds was demonstrated in mangrove areas in India, where MA were quantified from methylamines [14, 15].

In the rainy season, methanol remained important, but the abundance of MA from hydrogen and acetate increased under low sulfate concentrations, hydrogen theoretically contributes 33% to total methanogenesis when carbohydrates or similar organic matter are degraded, being important in environments with high sedimentation rates (*≈*10 cm/year) and organic carbon supplementation [36]. In the coastal lagoons studied, a high concentration of organic carbon (3.4–14.5%) was quantified, and a sedimentation rate of 6 cm/year was observed in Carretas-Pereyra. Acetate can produce approximately two thirds of total methane in freshwater sediments; however, its contribution to methane formation decreases when is consumed in other anaerobic processes as the sulfate reduction [4]. The effect of sulfate on methanogenesis was demonstrated in temperate estuaries, where the contribution of acetate for this process has been found to increase when sulfate concentration is low in freshwater zone, and the sulfate reduction decreased [7, 13]. The acetate and hydrogen are also important substrates for methanogenesis in salt marshes areas [10].

This study has revealed that acetate-utilizing and hydrogen-utilizing MA does not have a distinct vertical distribution pattern in Chantuto-Panzacola and Carretas-Pereyra sediments, whereas the methanol-based group apparently being more abundant in the 6–12 cm layer (*P* = 0.05). The presence of MA along 12 cm of sediment column seems to be a result of the availability of substrates for these microorganisms; the constant supply of different substrates favors the presence of MA at different sediment layers as also has been demonstrated in sediments of tidal flats, coastal marshes, and mangroves [8, 10, 14]. 

### 3.3. Acetoclastic Metabolic Activity

In all kinetic experiments, there was an increase in the concentration of acetate in the first days, along with other volatile fatty acids (propionate and butyrate); this pattern reveals the presence of fermentation processes in sediments. The continued presence of acetate along with other intermediaries (butyrate and propionate) is similar to that reported in other studies where methanogenesis has been assessed [37]. Acetate is an important intermediate produced during the anaerobic mineralization of organic matter, followed by propionate and other volatile fatty acids [38]. The fermentation activity is important because it releases organic substrates, such as acetate, that can be used by the MA, which cannot directly use complex organic compounds. Subsequent to the production of volatile fatty acids, acetate consumption started on day 7 in sulfate-enriched media and between days 14 and 21 in sulfate-free media. Methane production was recorded on day 21.

**Figure 3:**
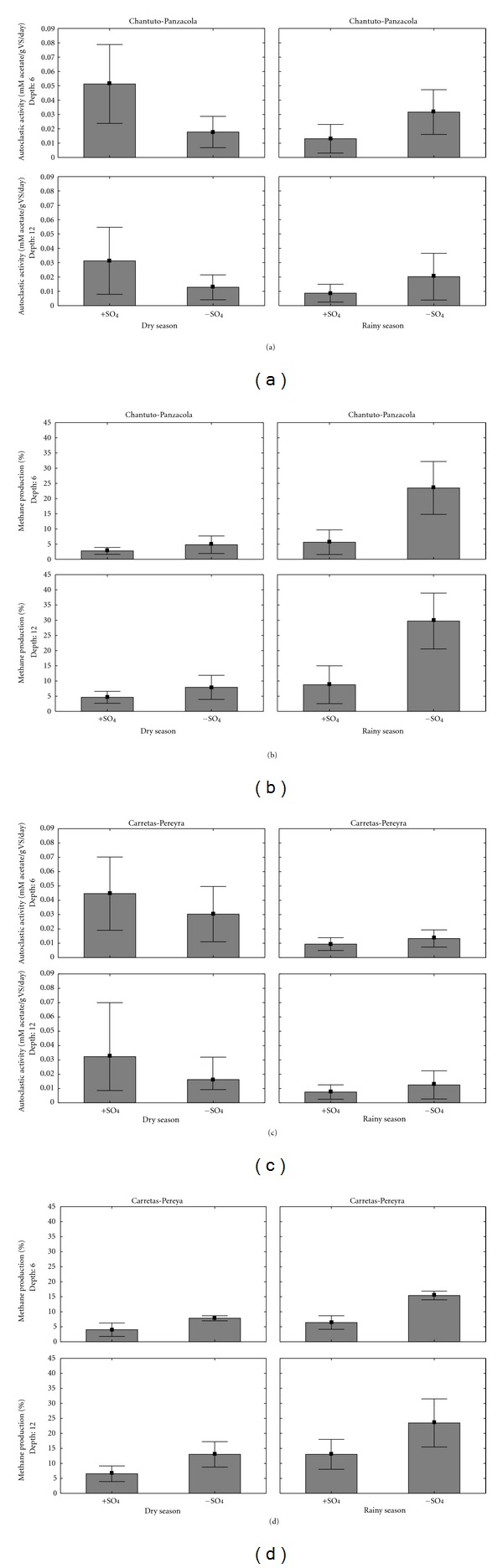
Temporal and spatial variations of acetoclastic activity and methane production in Chantuto-Panzacola (a, b) and Carretas-Pereyra (c, d).

Acetoclastic activity in sulfate-free experiments had no significant temporary differences (*P* > 0.05) (Figures 3(a)–3(c)). The experiments with sulfate showed significant temporal fluctuations, with high values in the dry season (Table 3; Figures 3(a)–3(c)). Vertical variations did not reach statistical significance (*P* > 0.05).

Methane formation was observed in all experiments, with differences depending on the specific conditions of each medium. The addition of acetate results in an increase in methane production in relation to the amount observed in controls (no carbon supplementation). 

Methane production was higher in sulfate-free media compared with sulfate-enriched media (Table 3; Figures 3(b)–3(d)). Temporal differences (*P* < 0.05) in methane production from acetate were observed in both systems. Methane levels were higher in the rainy season than in the dry season (Table 3). Significant vertical changes (*P* < 0.05) were observed only in Carretas-Pereyra: a lower production in the upper 6 cm and a higher methane production in the 6–12 cm layer (Table 3, Figures 3(b)–3(d)). 

The presence of sulfate in the culture media influenced methanogenic activity. In the sulfate-free experiments a peak of acetoclastic activity was observed coupled with a rise in methane production in sediments during the rainy season and in the deep layer, suggesting that methanogenesis was favored. Studies demonstrated that potential methanogenesis from acetate was higher in the absence of sulfates [37]. By contrast, the addition of sulfate resulted in an increase of acetoclastic activity in the dry months and in the upper sediment layer, and methane production declined. In sediments of coastal lagoons and mangrove areas in India, an increase in the production and emission of methane was determined in freshwater areas compared to brackish regions. Also, methane emissions were higher in the postmonsoon season, when salinity and sulfate concentration were lower [16, 34].

### 3.4. Environmental Variables and MA

**Figure 4:**
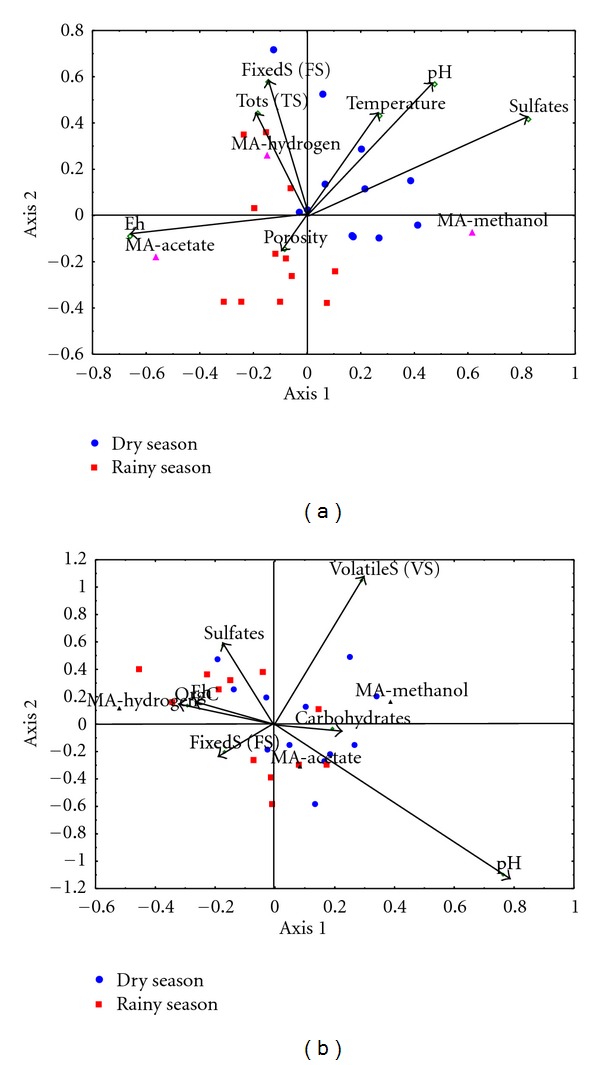
Relationship between environmental variables and methanogenic community in Chantuto-Panzacola (a) and Carretas-Pereyra (b).

The correlation coefficients between environmental variables and ordination axes (interset correlation) obtained by CCA denote the relative importance of each environmental variable in the distribution of the methanogenic community. For Chantuto-Panzacola, the MA-environment correlation was 0.92 corresponded to a salinity-sulfate gradient and 0.60 for pH. CCA results for Carretas-Pereyra showed a correlation of 0.74 for pH and volatile solids, and 0.43 for volatile solids. The ordination diagram obtained by CCA showed a change in the structure of the methanogenic community with regard to certain environmental variables (Figure 4). The first axis accounted for 65.62% of total variance in Chantuto-Panzacola, corresponding to a salinity-sulfate gradient (Figure 4(a)). In the right side of the diagram, those sites with the highest sulfate concentration, temperature, and pH (dry season) were grouped, in these conditions methanol-utilizing MA were abundant. The left side of the plot-grouped sites with highest total solids content where hydrogen-utilizing MA prospered, whereas acetate-utilizing MA abound in sites with a higher porosity and less reduced conditions (Figure 4(a)). In Carretas-Pereyra, to the plot's upper left side, the first axis accounted for 29.08% of variance and salinity-sulfate, Eh and organic carbon concentration were all correlated with hydrogen-utilizing MA abundance, mainly during the rainy season. Abundance of methanol-utilizing MA was correlated with volatile solids during the dry season and acetate-utilizing MA prospered in a pH gradient in both seasons.

The presence of sulfate significantly influenced the abundance of MA in both systems. In sulfate-rich conditions the anaerobic process that is most favored is the sulfate reduction. Elevated levels of SRP in the dry season (10^8^–10^10^ cells/g) and decrease in the rainy season (10^5^–10^7^ cells/g) quantified in coastal lagoons studied support this hypothesis [39]. The relationship between MA and sulfate content is consistent with knowledge on these communities in sediments of temperate estuaries, tropical coastal lagoons, coastal marshes, and mangroves [7–10, 13, 14, 16].

The temperature, pH, Eh, and organic fractions were other variables contributing to the presence of MA. In this study the bacteria grew in a temperature range of 26.7–29.4°C, the optimum temperature reported for the development of methanogens is 30–32°C in tropical wetlands, whereas in mangrove sediments MA have been observed at temperature ranges between 26 and 30°C [14, 15]. The pH conditions (6.7–7.1) were favorable for methanogenic community. Mohanraju and Natarajan [15] associated the presence of MA with pH values of 6.6–7.2 in mangrove sediments, whereas in coastal marshes sediments MA were recorded in pH ranges of 6.1–7.5 [10]. The reducing (−100 to +100 mV) and highly reducing (−300 to −100 mV) characteristics of sediment also contributed to MA development, these have been reported at levels from Eh below −150 mV in coastal marshes [40]. 

## 4. Conclusions

**Figure 5:**
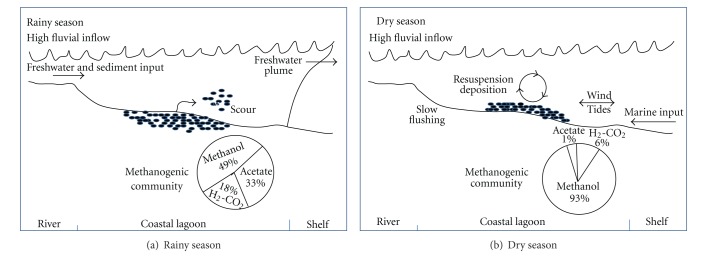
Conceptual model on MA dynamics in sediments for tropical coastal lagoons.

The MA was a constant component involved in anaerobic mineralization of organic matter in the sediments of the coastal systems Chantuto-Panzacola and Carretas-Pereyra. Their populations were active by its ability to utilize different substrates, especially methanol. In these ecosystems, changes in precipitation and its influence on fluvial inputs significantly impacted salinity and sulfate content, which was the main factor regulating the temporal dynamics of methanogenic community. In the rainy season, the increase in river inflow to lagoons produces freshwater conditions, low sulfate concentrations, sediment resuspension, and less reducing redox potentials. The environmental characteristics that prevail in this season lead to an increase in MA abundance, with the following decreasing rank by substrate: methanol > H_2_-CO_2_ > acetate (Figure 5(a)). Methanogenic activity from acetate is higher and results in a rise in methane production. The peak of methanogenic activity in the rainy season suggests that these ecosystems may be an important source of atmospheric CH_4_ and CO_2_ in this season. In the dry season, the freshwater inflow declines and salinity, sulfate, and inorganic solids content increases, along with more negative redox conditions. In this conditions a lower MA density was observed (Figure 5(b)), with the following order by substrate: methanol > acetate > H_2_-CO_2_. Our hypothesis is that this mechanism is cyclic and is controlled by changes associated to the seasonal fluctuations in fluvial inflow and precipitation. We considered that this model could be applied to other coastal lagoons and wetlands (mangroves) in tropical latitudes, characterized by a high organic matter concentration and a permanent river discharge, with significant seasonal variations in discharge volume. These characteristics favor the continued presence of the MA in tropical coastal systems and control their temporal dynamics. In estuaries, the absence of barriers that restrict communication with the sea difficult to apply this model because the tidal influence decreases the impact of freshwater input. In these systems the freshwater influence is more important at spatial level.

## Figures and Tables

**Table 1:** Environmental variables in the coastal lagoon sediments of Chantuto-Panzacola and Carretas-Pereyra, Chiapas. Mean ± Standard deviation.

	Dry season		Rainy season	
Depth	6	12	6	12
Chantuto-Panzacola				

Temperature (°C)	29.2 ± 1.1	28.3 ± 1.3	28.1 ± 1.5	26.7 ± 1.5
Salinity (‰)	21.3 ± 6.1	18.6 ± 5.1	2.5 ± 2.5	2.8 ± 3.1
Sulphate (mM)	11.0 ± 1.8	9.8 ± 1.2	3.8 ± 1.4	2.9 ± 0.9
pH	7.1 ± 0.1	7.0 ± 0.1	6.7 ± 0.2	6.8 ± 0.1
Eh (mV)	−206 ± 76	−356 ± 34	−104 ± 4	−286 ± 53
Total Solids (TS, g/L)	445.50 ± 120.65	338.12 ± 79.11	320.79 ± 153.2	303.50 ± 151.07
Volatile Solids (VS, g/L)	42.61 ± 20.19	47.40 ± 34.86	75.82 ± 41.0	68.76 ± 51.98
Porosity (g/cm^3^)	0.3 ± 0.1	0.4 ± 0.08	0.4 ± 0.1	0.4 ± 0.1
Organic matter (%)	7.2 ± 3.4	5.9 ± 3.8	9.8 ± 5.5	5.8 ± 3.2
Organic carbon (%)	4.1 ± 2.0	3.4 ± 2.2	5.7 ± 3.1	3.3 ± 1.8
Carbohydrates (mg/L)	5.6 ± 4.0	6.5 ± 4.0	5.0 ± 1.0	5.9 ± 4.1

Carretas-Pereyra				

Temperature (°C)	29.4 ± 0.8	28.5 ± 0.7	28.5 ± 1.9	28.3 ± 0.9
Salinity (‰)	27.3 ± 5.3	23.5 ± 3.3	4.3 ± 4.08	3.2 ± 3.8
Sulphate (mM)	13.0 ± 1.4	11.7 ± 1.3	3.5 ± 1.98	1.9 ± 1.4
pH	6.9 ± 0.1	6.8 ± 0.1	6.8 ± 0.1	6.7 ± 0.1
Eh (mV)	−296 ± 83	−411 ± 66	−152 ± 46	−369 ± 99
Total Solids (TS, g/L)	261.70 ± 135.49	229.49 ± 134.29	211.56 ± 123.36	188.41 ± 97.24
Volatile Solids (VS, g/L)	75.22 ± 35.27	85.05 ± 71.58	28.40 ± 12.90	40.39 ± 22.98
Porosity (g/cm^3^)	0.2 ± 0.07	0.2 ± 0.1	0.4 ± 0.1	0.5 ± 0.1
Organic matter (%)	12.5 ± 4.5	25.4 ± 19.2	10.0 ± 4.5	15.7 ± 8.8
Organic carbon (%)	7.2 ± 4.5	14.5 ± 11.02	6.1 ± 2.6	9.03 ± 5.04
Carbohydrates (mg/L)	6.8 ± 3.6	6.0 ± 3.5	3.8 ± 1.2	5.3 ± 3.5

**Table 2:** Results of the ANOVA (*F*) and multiple comparisons analysis (MCA) (Tukey test) of environmental and microbiological variables between seasons and sediment depth in Chantuto-Panzacola and Carretas-Pereyra. *P*: significance. Seasons: D: dry and R: rainy. Depth: 6 cm and 12 cm.

Variables	Season			Depth		
	*F *	*P *	MCA	*F *	*P *	MCA
Chantuto-Panzacola						

Temperature (°C)	4.66	0.0421	D > R	3.75	0.0684	—
Salinity (‰)	62.03	0.0000	D > R	0.08	0.9311	—
Sulphate (mM)	109.00	0.0000	D > R	0.45	0.5349	—
pH	28.81	0.0000	D > R	0.01	0.9427	—
Eh (mV)	4.54	0.0446	D < R	38.04	0.0000	6 < 12
MA-Acetate (cells/g)	112.38	0.0000	D < R	0.01	0.7842	—
MA-Hydrogen (cells/g)	15.10	0.0008	D < R	0.05	0.8195	—
MA-Methanol (cells/g)	5.92	0.0236	D < R	3.36	0.0528	—
Activity + SO_4_ (mM acetate/g VS/day)	14.71	0.0009	D > R	1.50	0.2321	—
CH_4_ + SO_4_	66.12	0.0000	D < R	0.85	0.3085	—
CH_4_ − SO_4_	4.96	0.0364	D < R	2.17	0.0831	—

Carretas-Pereyra						

Temperature (°C)	0.97	0.3344		1.28	0.2705	—
Salinity (‰)	154.47	0.0000	D > R	0.26	0.6156	—
Sulphate (mM)	210.03	0.0000	D > R	0.45	0.5101	—
pH	10.47	0.0038	D > R	1.09	0.3088	—
Eh (mV)	3.80	0.0641	—	19.80	0.0002	6 < 12
MA-Acetate (cells/g)	4.82	0.0390	D < R	0.13	0.7193	—
MA-Hydrogen (cells/g)	9.39	0.0057	D < R	0.48	0.4952	—
MA-Methanol (cells/g)	2.71	0.1142	—	1.06	0.3142	—
Activity + SO_4_ (mM acetate/g VS/day)	12.62	0.0018	D > R	0.46	0.5042	—
CH_4_ + SO_4_	15.39	0.0007	D < R	6.24	0.0204	6 < 12
CH_4_ − SO_4_	7.21	0.0135	D < R	7.88	0.0103	6 < 12

**Table 3:** Abundance of MA, acetoclastic activity, and methane production in sediments of Chantuto-Panzacola and Carretas-Pereyra, Chiapas. Mean values.

	Dry season		Rainy season	
Depth	6	12	6	12
Chantuto-Panzacola				

MA-acetate (cells/g)	1.30 × 10^5^	2.99 × 10^4^	2.20 × 10^7^	2.09 × 10^7^
MA-Hydrogen (cells/g)	1.63 × 10^6^	9.55 × 10^4^	9.37 × 10^6^	8.63 × 10^6^
MA-methanol (cells/g)	1.79 × 10^6^	1.97 × 10^7^	1.17 × 10^7^	2.06 × 10^7^
Acetate activity without SO_4_^−2^ (mM acetate/g VS/day)	0.03	0.03	0.02	0.01
Acetate activity with SO_4_^−2^ (mM acetate/g VS/day)	0.05	0.03	0.01	0.01
% CH_4_ without SO_4_^−2^	4.81	7.91	23.50	29.73
% CH_4_ with SO_4_^−2^	2.78	4.63	5.64	8.77

Carretas-Pereyra				

MA-acetate (cells/g)	4.52 × 10^4^	6.17 × 10^4^	1.90 × 10^6^	1.32 × 10^6^
MA-Hydrogen (cells/g)	1.34 × 10^5^	1.51 × 10^5^	4.49 × 10^6^	2.64 × 10^6^
MA-methanol (cells/g)	1.34 × 10^6^	8.24 × 10^6^	1.27 × 10^7^	2.21 × 10^7^
Acetate activity without SO_4_^−2^ (mM acetate/g VS/day)	0.03	0.02	0.01	0.01
Acetate activity with SO_4_^−2^ (mM acetate/g VS/day)	0.04	0.03	0.01	0.01
% CH_4_ without SO_4_^−2^	7.83	13.01	15.42	23.47
% CH_4_ with SO_4_^−2^	4.02	6.55	6.41	13.02
